# Maternal Ambient Exposure to Atmospheric Pollutants during Pregnancy and Offspring Term Birth Weight in the Nationwide ELFE Cohort

**DOI:** 10.3390/ijerph18115806

**Published:** 2021-05-28

**Authors:** Marion Ouidir, Emie Seyve, Emmanuel Rivière, Julien Bernard, Marie Cheminat, Jérôme Cortinovis, François Ducroz, Fabrice Dugay, Agnès Hulin, Itai Kloog, Anne Laborie, Ludivine Launay, Laure Malherbe, Pierre-Yves Robic, Joel Schwartz, Valérie Siroux, Jonathan Virga, Cécile Zaros, Marie-Aline Charles, Rémy Slama, Johanna Lepeule

**Affiliations:** 1 Univ. Grenoble Alpes, Inserm, CNRS, IAB, 38000 Grenoble, France; emie.seyve@univ-grenoble-alpes.fr (E.S.); valerie.siroux@univ-grenoble-alpes.fr (V.S.); remy.slama@univ-grenoble-alpes.fr (R.S.); johanna.lepeule@univ-grenoble-alpes.fr (J.L.); 2 Epidemiology Branch, Division of Intramural Population Health Research, Eunice Kennedy Shriver National Institute of Child Health and Human Development, Bethesda, MD 20892, USA; 3 ASPA, ATMO Grand Est, 67300 Schiltigheim, France; emmanuel.riviere@atmo-grandest.eu (E.R.); julien.bernard@atmo-grandest.eu (J.B.); 4 Ined-Inserm-EFS Joint Unit ELFE, 75020 Paris, France; marie.cheminat@ined.fr (M.C.); cecile.zaros@ined.fr (C.Z.); marie-aline.charles@inserm.fr (M.-A.C.); 5 ATMO Normandie, 76000 Rouen, France; jerome.cortinovis@atmonormandie.fr; 6 AIR Pays-de-la-Loire, 44300 Nantes, France; ducroz@airpl.org; 7 AIRPARIF, 75004 Paris, France; fabrice.dugay@airparif.fr; 8 ATMO Nouvelle-Aquitaine, 33000 Bordeaux, France; ahulin@atmo-na.org; 9 Department of Geography and Environmental Development, Ben-Gurion University of the Negev, Beer Sheva P.O. Box 653, Israel; ikloog@gmail.com; 10 ATMO France, 75004 Paris, France; anne.laborie@atmo-france.org; 11 U1086 INSERM-UCN ‘Anticipe’, 14000 Caen, France; ludivine.launay@inserm.fr; 12 National Institute for Industrial Environment and Risks (INERIS), 60550 Verneuil en Halatte, France; laure.malherbe@ineris.fr; 13 ATMO Occitanie, 31330 Toulouse, France; pierre-yves.robic@atmo-occitanie.org; 14 Exposure, Epidemiology, and Risk Program, Department of Environmental Health, Harvard T.H. Chan School of Public Health, Boston, MA 02115, USA; joel@hsph.harvard.edu; 15 ATMO PACA, 13006 Marseille, France; jonathan.virga@atmopaca.org; 16 Inserm Univ. Paris Descartes, U1153 CRESS, 75004 Paris, France

**Keywords:** air pollution, term birth weight, spatial autocorrelation, propensity score, doubly robust

## Abstract

Background: Studies have reported associations between maternal exposure to atmospheric pollution and lower birth weight. However, the evidence is not consistent and uncertainties remain. We used advanced statistical approaches to robustly estimate the association of atmospheric pollutant exposure during specific pregnancy time windows with term birth weight (TBW) in a nationwide study. Methods: Among 13,334 women from the French Longitudinal Study of Children (ELFE) cohort, exposures to PM_2.5_, PM_10_ (particles < 2.5 µm and <10 µm) and NO_2_ (nitrogen dioxide) were estimated using a fine spatio-temporal exposure model. We used inverse probability scores and doubly robust methods in generalized additive models accounting for spatial autocorrelation to study the association of such exposures with TBW. Results: First trimester exposures were associated with an increased TBW. Second trimester exposures were associated with a decreased TBW by 17.1 g (95% CI, −26.8, −7.3) and by 18.0 g (−26.6, −9.4) for each 5 µg/m^3^ increase in PM_2.5_ and PM_10_, respectively, and by 15.9 g (−27.6, −4.2) for each 10 µg/m^3^ increase in NO_2_. Third trimester exposures (truncated at 37 gestational weeks) were associated with a decreased TBW by 48.1 g (−58.1, −38.0) for PM_2.5_, 38.1 g (−46.7, −29.6) for PM_10_ and 14.7 g (−25.3, −4.0) for NO_2_. Effects of pollutants on TBW were larger in rural areas. Conclusions: Our results support an adverse effect of air pollutant exposure on TBW. We highlighted a larger effect of air pollutants on TBW among women living in rural areas compared to women living in urban areas.

## 1. Introduction

A large body of literature has been published in the last 20 years about the relationship between prenatal exposure to air pollutants and intrauterine growth of the fetus, most often evaluated by birth weight-related outcomes [1,2,3,4,5]. Rural and urban areas are characterized by different levels and mixtures of air pollutants and different gradients in social deprivation [6,7]. However, most studies have focused on urban areas [8,9,10], with a few exceptions [11,12,13]. Urbanization level and social deprivation are also associated with birth weight [14,15].

As part of its integrated science assessment, the EPA classifies the evidence of association between air pollution and birth weight as suggestive of a causal relationship for PM, and as suggestive but not sufficient to infer a causal relationship for NO_2_ [16,17]. In fact, although there has been an abundant literature on this issue [5,18,19,20,21], there is high heterogeneity in the results [18]. Such heterogeneity may be due to the methods used to quantify exposures to air pollutants that are more or less prone to spatial misalignment between participants’ location and the exposure estimate location [3]. It may also arise from the heterogeneity of outcome metrics used to define fetal growth (continuous (term) birth weight, dichotomized birth weight for all births or term births only, small for gestational age, etc.), the definition of exposure windows [22], the lack of confounders and the statistical methods used to account for confounding. Propensity score methods have been proposed as an effective way to account for confounding by reconstructing counterfactuals and mimicking a randomized experiment using observational data [23,24]. Although this approach is increasingly used in epidemiological studies, its application in the field of air pollution effects on birth outcomes is scarce. So far, only one study has modeled air pollution effects on birth weight using a causal modeling framework [25].

We sought to use stabilized inverse probability scores and doubly robust methods to investigate the association between atmospheric pollutant exposure during short- and long-term time windows and term birth weight (TBW) in a nationwide study. We studied term birth weight to overcome the influence of gestational duration and focus on fetal growth. We further examined whether this association was modified by urbanization and social deprivation.

## 2. Materials and Methods

### 2.1. Study Population

We relied on the French Longitudinal Study of Children (ELFE) cohort, which included 18,329 mother-child couples enrolled from a random sample of 349 maternity wards out of 540 in mainland France during four periods of 2011: 1 to 4 April; 27 June to 4 July; 27 September to 4 October; 28 November to 5 December. Mothers had to read French, Arabic, Turkish or English to be eligible. Mothers younger than 18 years, deliveries before 33 weeks of gestation (very preterm births) and multiple births of three children or more were excluded [26]. Informed consents were signed by the parents or the mother alone, allowing for the father being informed of his right to oppose participation. Individual data were collected by interview at birth. We restricted the present study to full-term babies to focus on the specific question of intrauterine growth. The cohort was approved by the relevant ethical committees (CNIL, Commission nationale de l’informatique et des libertés; CCTIRS, Comité Consultatif sur le traitement de l’Information en matière de Recherche dans le domaine de la Santé; CNIS, Conseil National de l’Information Statistique).

### 2.2. Maternal Exposure

Maternal exposure to outdoor PM_2.5_ (particulate matter with an aerodynamic diameter less than 2.5 µm), PM_10_ (particulate matter with an aerodynamic diameter less than 10 µm) and NO_2_ (nitrogen dioxide) was estimated at the geocoded maternal home addresses during pregnancy using nationwide models with fine spatial resolution (1 km grid) and daily temporal resolution (see Supplementary Materials of Benmerad et al. 2017 for further information) [27]. The exposure models used kriging methods to combine data from the CHIMERE chemistry-transport model [28,29] and measurements from air quality monitoring stations. The precision of kriging was evaluated through leave-one-out and k-fold cross-validation, and performance indicators of the model (bias, mean absolute normalized error, root-mean-square error and correlation) were computed. Median correlations between estimated (leave-one-out cross-validation) and measured average concentrations were 0.93 (25th–75th percentiles, 0.90–0.96) for PM_2.5_, 0.93 (0.88–0.95) for PM_10_ and 0.90 (0.84–0.93) for NO_2_.

Maternal exposures were averaged from daily exposure for the whole pregnancy (conception to delivery), for each trimester (92 days if no delivery) of pregnancy and for the 30, 60 and 90 days before delivery. Since exposure during the 3rd trimester of pregnancy is influenced by the length of the gestation, we also considered exposure during the 3rd trimester truncated at the end of the 37th gestational week. The conception date was estimated as the 14th day after the start of the last menstrual period [30]. When the discrepancy between conception date calculated from the last menstrual period dating and from the ultrasound dating was 10 days or more, we used ultrasound-based estimates. 

### 2.3. Contextual Characteristics

Social deprivation was estimated by the European deprivation index (EDI) available at the Ilots Regroupés pour l’Information Statistique (IRIS) level [31,32]. IRIS is the smallest geographical census unit in France; each IRIS includes approximately 2000 individuals with relatively homogeneous social characteristics. The EDI was categorized in quintiles, the first quintile representing the least deprived areas, and the fifth quintile representing the most deprived areas [7].

Urbanization level as defined by the National Institute of Statistics and Economic Studies (INSEE) of towns has 3 groups: city centers, suburban areas and rural areas. In our study, the residential urbanization level was categorized as follows: large city centers (≥100,000 inhabitants), suburban areas and small city-center areas (<100,000 inhabitants) and rural areas.

### 2.4. Regression Models

We used inverse probability weighting and doubly robust methods [33,34] to estimate effects of prenatal exposure to air pollutants on TBW. The main analysis investigating the relationship between air pollution exposures and TBW used weighted generalized additive models with a Gaussian distribution and identity link function. A smooth function of latitude and longitude of the home address was added to account for spatial autocorrelation (Moran’s index *p*-value < 0.001). The linearity of the relationship was graphically confirmed using diagnostic plots. Weights were calculated as follows. First, we estimated propensity scores on exposure by modeling air pollutant exposure as a function of the following covariates: maternal education (up to secondary school, vocational qualification, high school, university degree), in relationship status (yes/no), parity (0, 1, 2, more children), sex of the child, maternal active smoking during pregnancy (yes/no), social deprivation (in quintile), maternal French citizenship (yes/no), maternal height (linear), maternal age (restricted cubic spline with 3 knots) and weight before pregnancy (restricted cubic spline with 3 knots) (Equation (S1) in the Supplementary Materials). The coding of the covariates was selected using a method previously described [35]. Interactions between the aforementioned variables were retained when the statistical significance of the interaction term was below 0.20. 

Second, propensity scores were used to calculate stabilized inverse probability weights (Equation (S2) in the Supplementary Materials) [36]. Stabilized weights were used for continuous exposures. They create a pseudo-population of the same size as the original population with the mean of weights being one (Table S1). Compared to non-stabilized weights, stabilized weights provide a more precise health effect estimate [23]. Inverse probability weighting based on the propensity score creates a pseudo-population where the exposure is independent of covariates. It allows us to reconstruct counterfactuals and mimics a randomized experiment using observational datasets. Consequently, the estimated difference in TBW associated with air pollution exposure is expected to be unbiased, provided that no important confounder was omitted. Including in the propensity model variables highly associated with exposure may result in an increased imprecision of the exposure effect [37,38]. In this study, air pollution exposure was highly explained by the season of conception and the urbanization level [7]. For this reason, urbanization level and season of conception were not included in the propensity score model, but only in the outcome regression models (Equation (S3) in the Supplementary Materials). We conducted several sensitivity analyses on this approach. First, we trimmed weights higher (respectively, lower) than the 99th (1st) percentile to the value at the 99th (1st) percentile [39]. Second, we changed covariates in the propensity models: 1) although adjustment for gestational duration has been discussed [40,41], it is a strong predictor of birth weight; we therefore added the gestational duration (restricted cubic spline) in the propensity models (Equation (S4) in the Supplementary Materials); (2) we identified maternal education, social deprivation, maternal French citizenship, parity and season of conception as the minimal sufficient adjustment set of covariates necessary to control for confounding [42,43]; thus, we conducted a sensitivity analysis using maternal education, social deprivation, maternal French citizenship and parity in the propensity models and further adjusted for season of conception (Equation (S5) in the Supplementary Materials).

After the sensitivity analyses, we moved to step 2 using the doubly robust method, which further adjusted the weighted outcome models on covariates included in the propensity models and season of conception (Equation (S6) in the Supplementary Materials). The doubly robust method gives two chances to correct for confounders the association between the exposure and the outcome; therefore, if at least one adjustment method is correctly specified, the effect estimate is unbiased [34]. In order to identify the specific effect of exposure during each trimester of pregnancy, we applied a method previously described by Bell et al., incorporating residuals of the regression between trimesters of exposure in the outcome models [44]; this approach accounts for the correlation of exposure among trimesters of pregnancy. Finally, we added interaction terms in the doubly robust regressions to investigate whether the effect of air pollutants on TBW differs by urbanization levels and social deprivation. A *p*-value < 0.05 was considered statistically significant. Analyses were performed with STATA 12 (StataCorp. 2011. Stata Statistical Software: Release 12. College Station, TX, USA) and R software (R-version 3.3.1 GUI) using the ‘mgcv’ package for GAMs [45].

## 3. Results

Among the 18,329 women from the ELFE cohort, we excluded 971 preterm births, 364 women with a multiple pregnancy, 93 women with congenital cytomegalovirus infection during pregnancy, 1014 women without a geocoded address and 1312 women who had more than 25% of missing data for at least one exposure time window. We conducted a complete-case analysis by discarding 1241 women with missing data on covariates, leaving 13,334 women for the study (Table 1). Most women had a university degree (60%), were in a relationship (95%) and 20% smoked during pregnancy. About 30% of women lived in the most socially deprived areas and 28% lived in rural areas. The conception date was estimated with the last menstrual period for 8918 (67%) pregnant women and with ultrasound for 4416 (33%). Mean (±SD) gestational duration was 40.0 ± 1.2 gestational weeks and mean TBW was 3363 ± 450 g. Mean pregnancy air pollutants levels were 17.3 ± 2.9 µg/m^3^ for PM_2.5_, 24.5 ± 3.4 µg/m^3^ for PM_10_ and 20.0 ± 8.9 µg/m^3^ for NO_2_ (Table 2). Pearson’s correlation highlighted a seasonality pattern of the particles (Pearson’s correlations were −0.57 and −0.41 between the 1st and 3rd trimester truncated at 37 weeks for PM_2.5_ and PM_10_, respectively) while correlation for NO_2_ ranged from 0.42 to 0.99 (Figure S1). 

### 3.1. Main Analysis

**Figure 1:**
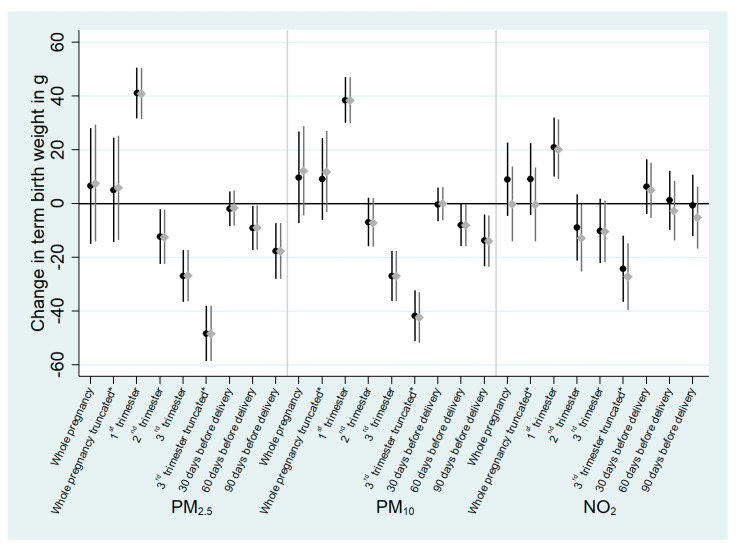
Association between atmospheric pollutant exposure during pregnancy and term birth weight (*n* = 13,334 pregnant women from ELFE cohort). All effect estimates correspond to an increase of 5 µg/m^3^ for PM_2.5_ and PM_10_ and 10 µg/m^3^ for NO_2_. Regression coefficients are from weighted generalized additive models^1^. Full weights are represented in black and trimmed weights in grey. The symbol reflects the central estimate, the line represents the 95% confidence interval. ^1^ using the stabilized inverse probability of being exposed to air pollutants calculated using maternal education, relationship status, parity, sex of the child, maternal active smoking during pregnancy, social deprivation, maternal French citizenship, maternal age, weight before pregnancy and maternal height, and further adjusted for urbanization level and season of conception. * exposure truncated at 37 gestational weeks.

There was no evidence of a significant association between TBW and average pregnancy maternal exposure for any of the pollutants (*p*-values above 0.2; Figure 1; Table S2). Exposure during the first trimester of pregnancy was associated with an increased TBW, while exposure during the second and third trimesters tended to be associated with a decrease in mean TBW. During the second trimester, each 5 µg/m^3^ increase in PM levels was associated with a reduction in mean TBW by 12.3 g (95% CI, −22.4, −2.2) for PM_2.5_ and 6.9 g (95% CI, −15.9, 2.1) for PM_10_. During the third trimester, a 5 µg/m^3^ increase in PM levels was associated with a decreased mean TBW of 27.0 g (95% CI, −36.6, −17.4) for PM_2.5_ and 26.9 g (95% CI, −36.6, −17.6) for PM_10_. To a lesser extent, NO_2_ levels during the second and third trimesters were associated with a reduction of mean TBW (−8.9 g; 95% CI, −21.3, 3.4, and −10.2 g; 95% CI, −22.2, 1.8, respectively). For all pollutants, the association between TBW and exposure during the third trimester was stronger when the third trimester exposure window was truncated at 37 gestational weeks: −48.3 g (95% CI, −58.6, −38.1), −41.7 g (95% CI, −51.2, −32.3) or −24.3 g (95% CI, −36.6, −12.1) for PM_2.5_, PM_10_ and NO_2_, respectively. Exposures during the last 60 and 90 days before delivery were associated with a decreased TBW for PM, while for NO_2_ no significant association was highlighted.

### 3.2. Sensitivity Analyses

Results of the sensitivity analysis with observations trimmed for extreme weights were similar to those of the main analysis for all exposure windows (Figure 1). Although most associations were still statistically significant, considering gestational duration in the propensity models shifted the associations between exposure to air pollutants and TBW toward the null (Figure S2). When we restricted the number of covariates included in the propensity models using the minimum set of confounders, the results did not differ from those of the main analysis (Figure S3).

### 3.3. Doubly Robust GAM

**Figure 2:**
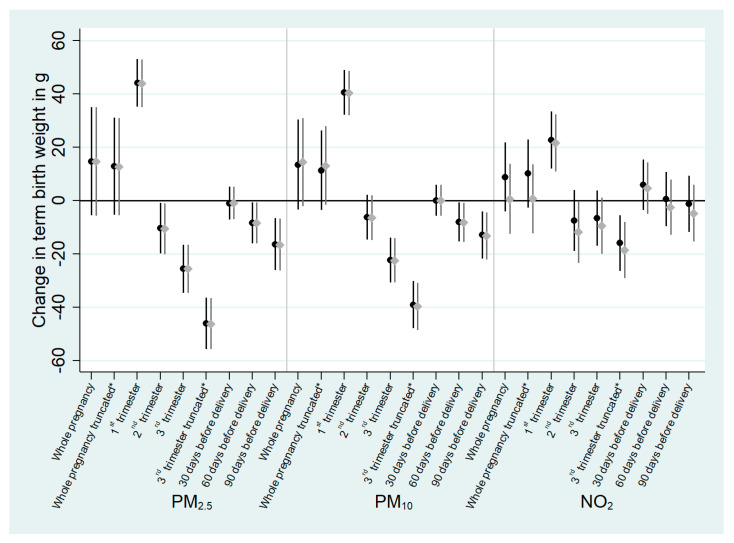
Adjusted difference in mean term birth weight (g) associated with atmospheric pollutant exposures during pregnancy using the doubly robust method (*n* = 13,334 pregnant women from ELFE cohort). All effect estimates correspond to an increase of 5 µg/m^3^ for PM_2.5_ and PM_10_ and 10 µg/m^3^ for NO_2_. Regression coefficients are from weighted doubly robust generalized additive models ^1^. Estimates corresponding to models with full weights (in black) and trimmed weights (in grey) are represented. The point shows the central estimate, the line represents the 95% confidence interval. ^1^ using the stabilized inverse probability of being exposed to air pollutants calculated using maternal education, in relationship status, parity, sex of the child, maternal active smoking during pregnancy, social deprivation, maternal French citizenship, maternal age, weight before pregnancy and maternal height, and further adjusted for the aforementioned covariates plus urbanization level and season of conception. * exposure truncated at 37 gestational weeks.

**Figure 3:**
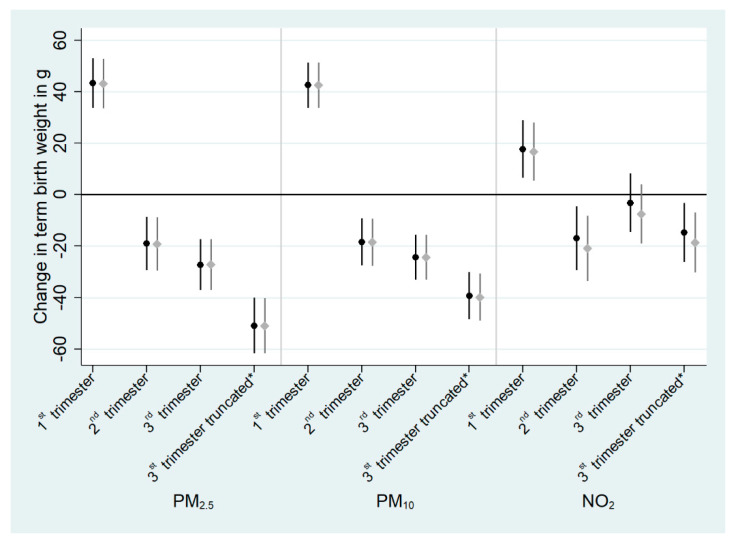
Adjusted difference in mean term birth weight (g) associated with atmospheric pollutant exposures during pregnancy (*n* = 13,334 pregnant women from ELFE cohort) accounting for the correlation among trimesters of pregnancy. All effect estimates correspond to an increase of 5 µg/m^3^ for PM_2.5_ and PM_10_ and 10 µg/m^3^ for NO_2_. Regression coefficients are from weighted doubly robust generalized additive models ^1^. Estimates corresponding to models with full weights (in black) and trimmed weights (in grey) are represented. The point shows the central estimate, the line represents the 95% confidence interval. ^1^ using the stabilized inverse probability of being exposed to air pollutants calculated using maternal education, in relationship status, parity, sex of the child, maternal active smoking during pregnancy, social deprivation, maternal French citizenship, maternal age, weight before pregnancy and maternal height, and further adjusted for the aforementioned covariates plus urbanization level, season of conception and residuals of the regression between trimesters. * exposure truncated at 37 gestational weeks.

Using the doubly robust method did not change the results of the association between pollutant exposure and TBW (Figure 2, Table S3). Accounting for correlation of exposures among trimesters of pregnancy in the doubly robust models did not change the results (Figure 3, Table S4). In the trimester-specific analyses, the first trimester exposure was significantly associated with an increased mean TBW (by 45.9 g; 95% CI, 36.9, 54.9 for PM_2.5_; by 42.3 g, 95% CI, 34.0, 50.6 for PM_10_; and by 18.4 g, 95% CI, 7.6, 29.1 for NO_2_), the second trimester exposure was significantly associated with a decreased mean TBW (by 17.1 g; 95% CI, −26.8, −7.3 for PM_2.5_; by 18.0 g; 95% CI, −26.6, −9.4 for PM_10_; and by 15.9 g; 95% CI, −27.6, −4.2 for NO_2_) and the third trimester exposures truncated at 37 gestational weeks were significantly associated with a decreased mean TBW (by 48.1 g; 95% CI, −58.1, −38.0 for PM_2.5_; by 38.1 g; 95% CI, −46.7, −29.6 for PM_10_; and by 14.7 g; 95% CI, −25.3, −4.0 for NO_2_).

The *p*-values of interaction terms between air pollutants and the urbanization level were generally lower than 0.2, in favor of suggesting a difference in the association between exposure and TBW across urbanization levels (Table 3). Estimated effects of air pollutants on TBW tended to be larger in rural areas compared to large city-center and suburban areas, the third trimester exposures truncated at 37 gestational weeks were significantly associated with a decreased mean TBW by 30.0 g (95% CI, −45.6, −14.5) in large city-center areas, by 45.6 g (95% CI, −56.4, −34.8) in suburban areas and by −69.3 g (95% CI, −45.6, −14.5) in rural areas for PM_2.5_; patterns were similar for the three pollutants. Regarding social deprivation, the results suggested an effect measure modification by social deprivation of the association between term birth weight and PM_2.5_ exposure toward the end of the pregnancy and PM_10_ and NO_2_ exposure average during pregnancy (*p*-values of interaction <0.2, Table S5). The significant negative association between PM_2.5_ exposure during the third trimester of pregnancy and TBW tended to be larger in the least deprived areas. We observed a significant positive association between averaged PM_10_ exposure during pregnancy and TBW in the least deprived areas.

## 4. Discussion

We showed that PM_2.5_, PM_10_ and NO_2_ exposure during the second and third trimester truncated at 37 GW was significantly associated with a reduction of TBW, while accounting for spatial autocorrelation and correlation between the three trimesters of exposures. In agreement with results observed for PM exposure during the third trimester, short-term exposure windows (60 and 90 days) before delivery were inversely associated with TBW, while no association was highlighted for NO_2_. Maternal exposure to PM and NO_2_ during the first trimester of pregnancy was significantly associated with an increased TBW, which was not expected. Our results further suggest that inverse associations between air pollutant exposure and TBW are larger in rural areas. Such associations may also vary by social deprivation levels.

This study has several strengths. We used GAM to account for spatial autocorrelation. Using ordinary regression with spatially autocorrelated data would produce residuals with spatial dependence, which could bias the results [46]. Although the use of GAM could lead to an attenuation of the magnitude in the association [47,48], our results on 13,334 women showed significant relationships between air pollutant levels and mean TBW. Furthermore, the doubly robust method gave us two chances to correctly adjust our associations. Our results remained consistent among different model specifications, which indicates a relevant selection of the adjustment variables [34,49]. Controlling for gestational duration in studies on environmental effects on offspring birth weight has been discussed and deemed relevant [40,41]. In our study, adjustment for gestational duration gave slightly smaller estimates of the relationship between air pollutants and TBW. A study conducted in Canada also reported smaller effects estimates when adjusting for gestational duration [13]. Adjustment for the smallest set of covariates did not change our conclusions. Even if unnecessary adjustment does not modify the average effect, the precision of the estimates can be affected. Therefore, decreasing the number of adjustment variables is interesting to explore [50]. On the other hand, in the context of measurement error in confounders (and hence possible residual confounding), adjusting for more than the minimum set of potential confounders can be seen as a robust approach. Further, our findings are important to consider in relation to studies that did not adjust for maternal tobacco use or gestational duration because this information was not available. We highlighted, in line with previous studies, that controlling for maternal education may partially take into account the smoking status [51,52,53].

Previous studies on birth weight have been relatively inconsistent as to which time period is the most critical regarding air pollution exposure. Since the total pregnancy and the third trimester duration differ among births, the length of the exposure windows might differ by 1 to 5 weeks (acknowledging that a pregnancy cannot go beyond 42 weeks) across births and possibly bias the results. To address this issue, we truncated the exposure during the 3rd trimester at 37 weeks for all births and explored shorter time windows focused on the end of pregnancy (30, 60 and 90 last days before birth). Health effects estimates were larger when the third trimester was truncated at 37 weeks, compared to the full third trimester. PM exposures during the last 30 days before birth were not significantly associated with TBW, while the last 60 and 90 days were negatively associated with TBW. Altogether, these results suggest that the window of vulnerability to air pollutants is rather in the first half of the third trimester. Our results accounted for the correlations that arise from investigating multiple time windows, further indicating a larger effect estimate during the third trimester than the second trimester.

Our results with a relatively low level of exposure on average (below the current European air quality standards) suggest a deleterious effect of air pollution exposure on TBW in accordance with a study conducted in USA relying on 61,640 women [54]. Our finding of a negative association between exposure to PM during the second and third trimesters and TBW is consistent with other studies [8,55,56,57]. For example, in Connecticut and Massachusetts, Bell et al. showed an adverse effect of PM_2.5_ during the second and third trimesters and PM_10_ during the third trimester on birth weight (reduction ranged from 7 to 9 g and 5 g to 7 g for an increase of 2.2 µg/m^3^ of PM_2.5_ and from 5 to 7 g for an increase of 7.4 µg/m^3^ of PM_10_) [44]. In this study, NO_2_ exposure during the first trimester of pregnancy was associated with a lower birth weight, which differs from our results. In Sweden, exposure to NO_x_ was not associated with birth weight and a risk of low birth weight; however, comparing the traffic density, which is highly correlated with urban/rural areas, in areas with more than 10 cars/min, the birth weights were lower than in areas with less traffic [11]. In Georgia, a study on 180,440 births relying on a PM_2.5_ monitor reported a reduction of 12 g in TBW for each increase of 5 µg/m^3^ of PM_2.5_ during the third trimester, while results on other trimesters were not significant [57]. As for PM_10_, Salam et al. indicated that a 5 µg/m^3^ increase in PM_10_ during the third trimester led to a 5 g lower TBW in 3901 children born in California, while an association that was close to the null was observed with PM_10_ exposure during the first trimester [8]. In northern Nevada, a study relying on 36,305 births showed a lower TBW of 5.5 g per 5 µg/m^3^ increase of PM_10_ during the third trimester, while other trimesters were not significantly associated with birth weight [55]. For comparison, our results suggest a lower TBW of 17 g and 18 g per 5 µg/m^3^ increase of PM_2.5_ and PM_10_ during the second trimester, respectively, and 25 g and 24 g during the third trimester, respectively. More broadly, our estimates of the association between trimester-specific exposure to PM_2.5_ and PM_10_ and TBW are similar in direction and larger in magnitude than those from most prior studies. One study conducted in Seoul found a higher birth weight of 4 g per 5 µg/m^3^ increase of PM_10_ during the first trimester [58]. Although our results also indicated a positive association between the first trimester exposure to air pollutants and TBW, there is no biological basis for such a positive association. Although some associations might be pure chance findings, these are not likely to explain the consistent and significant associations observed across the three pollutants. Other studies have reported prenatal exposure associated with a higher birth weight [10,12,59]. As for particles, this result may be due to the seasonality of PM, and so, if higher exposure during the 3rd trimester is deleterious to the birth weight, it may appear as an increase of exposure during the first trimester is associated with a higher birth weight (1st and 3rd trimester exposures were inversely correlated in our study). Finally, the endocrine disruptive function of the air pollutants has been proposed as a potential mechanism resulting in higher birth weight [60].

Our study is the first on this issue at the nationwide level in France. It includes a large sample size with geographic diversity. Most studies until now have focused on single metropolitan areas [12,59,61]. Two were conducted in Nancy and Poitiers relying on the EDEN cohort with around 1000 births: the study of Lepeule et al. pointed towards a deleterious effect of NO_2_ levels during the first trimester, while Sellier et al. found a positive association between birth weight and PM_10_ exposure during the second trimester of pregnancy [59,61]. The third study, relying on 3226 women in the Brittany region found a positive association between NO_2_ exposure during pregnancy and birth weight among newborns boys and no clear association among girls [12].

Although the geographical diversity in our study might have increased the potential for confounding, it also provided us with the opportunity to evaluate a large gradient of exposures to air pollutants in association with birth weight [6]. The inclusion in the analysis of women living in rural areas was possible thanks to the nationwide coverage of our exposure models. In our study, air pollutant exposures were higher in large city centers, compared with suburban areas and rural areas (results not shown, but previously published [2]). Our results highlighted a greater effect of air pollutants on TBW in rural areas compared to urban areas. This is consistent with results from another study in Brittany, France [12] and with a study in Canada [13]. This effect modification between urban and rural areas may be explained by a higher vulnerability of the populations living in rural areas, or by the different mixture of particles depending on the sources (e.g., wood smoke vs. traffic), which can lead to different toxicity [62]. Another explanation could be that our dispersion model with a 1 km grid did not have a sufficient resolution in cities where spatial variations could be high, and hence did not capture these variations, leading to increased measurement error in urban, compared to rural areas [63]. The fine spatio-temporal resolution of our exposure models allowed us to finely estimate outdoor exposure for long (whole pregnancy, trimesters) and shorter (30 and 60 days before delivery) time windows. It also allowed us to consider the residential mobility during pregnancy, which can lead to measurement error when not accounted for [64]. We assumed that outdoor exposure at home was similar to personal exposure. If this hypothesis does not hold, exposure misclassification can possibly lead to bias in the dose-response function [65,66].

Some studies hypothesized that air pollutant exposure could be more deleterious for the most socially deprived women. In our study, the effect of air pollution exposure by quintile of EDI gave different conclusions across pollutants. In the least deprived neighborhoods, PM_2.5_ tended to have a more deleterious effect during the end of the pregnancy, while average PM_10_ exposure during the pregnancy tended to be related to an increased TBW. In the literature, findings are inconsistent [67,68], including in France [69,70,71].

Several mechanisms have been proposed to explain the effects of exposure to air pollution on birth weight. Some of these hypotheses are linked to an alteration of the placental function with a lower placental weight or a modification of DNA methylation [72,73,74]. Studies also highlighted that air pollution could be associated with cardiovascular effects such as blood viscosity among men and women [75] as well as pregnancy-induced hypertension [76,77] that could alter materno-fetal exchanges, thus leading to a lower birth weight [78]. Oxidative stress and endocrine disruption have also been proposed as biological mechanisms between air pollution exposures and birth outcomes in animals, but have rarely been studied on humans [78].

## 5. Conclusions

Among a population of women with average exposures to PM_2.5_, PM_10_ and NO_2_ below the current European air quality standards of 25, 40 and 40 µg/m^3^, respectively, this nationwide study suggests an adverse effect of exposure to these pollutants during the second trimester and first half of the third trimester of pregnancy on the birth weight of the baby at term.

## Figures and Tables

**Table 1:** Characteristics of the whole ELFE cohort (*n* = 17,358), and of the subpopulations included (*n* = 13,334) and excluded (*n* = 4024) from this study (all groups were restricted to term births, our target population).

	Whole ELFE Population (*n* = 17,358)		Included Population (*n* = 13,334)		Excluded Population (*n* = 4024)		*p*-Value ^1^
	*n*	*n* (%) or Mean ± sd	*n*	*n* (%) or Mean ± sd	*n*	*n* (%) or Mean ± sd	
**Maternal age** (years)	17,186	30.6 ± 5.0	13,334	30.7 ± 5.0	3852	30.5 ± 5.1	0.03
**In relationship** (married, cohabitation)	17,100		13,334		3766		
Yes		16,177 (94.6)		12,608 (94.6)		3569 (94.8)	0.61
No		923 (5.4)		726 (5.4)		197 (5.2)	
**Maternal level of education**	17,191		13,334		3857		
Up to Secondary School		844 (4.9)		630 (4.7)		214 (5.5)	0.05
Vocational qualification		2384 (13.9)		1823 (13.7)		561 (14.5)	
High School		3586 (20.9)		2814 (21.1)		772 (20.0)	
University degree		10,377 (60.4)		8067 (60.5)		2310 (59.9)	
**Maternal French citizenship**	17,085		13,334		3751		
Yes		15,051 (88.1)		11,761 (88.2)		3290 (87.7)	0.41
No		2034 (11.9)		1573 (11.8)		461 (12.3)	
**Parity**	16,675		13,334		3341		
0		7887 (47.3)		6015 (45.1)		1872 (56.0)	<0.001
1 child		5596 (33.6)		4647 (34.9)		949 (28.4)	
2 children		2222 (13.3)		1876 (14.1)		346 (10.4)	
≥3 children		970 (5.8)		796 (6.0)		174 (5.2)	
**Maternal height** (cm)	17,191	165.0 ± 6.3	13,334	165.0 ± 6.3	3857	165.2 ± 6.3	0.07
**Maternal weight before pregnancy** (kg)	16,965	63.9 ± 13.6	13,334	64.0 ± 13.6	3631	63.9 ± 13.9	0.93
**Maternal active smoking during pregnancy**	17,054		13,334		4679		
Yes		3393 (19.9)		2615 (19.6)		1013 (21.6)	0.08
No		13,661 (80.1)		10,719 (80.4)		3666 (78.4)	
**Deprivation index** (EDI, quintile)	16,466		13,334		3132		
Q1 (least deprived)		2808 (17.1)		2287 (17.2)		521 (16.6)	0.35
Q2		2902 (17.6)		2383 (17.9)		519 (16.6)	
Q3		2688 (16.3)		2166 (16.2)		522 (16.7)	
Q4		3090 (18.8)		2499 (18.7)		591 (18.9)	
Q5 (most deprived)		4978 (30.2)		3999 (30.0)		979 (31.3)	
Urbanization level	16,469		13,334		3135		
City-center areas		2797 (17.0)		2266 (17.0)		531 (16.9)	0.003
Suburban areas		9148 (55.5)		7331 (55.0)		1817 (58.0)	
Rural areas		4524 (27.5)		3737 (28.0)		787 (25.1)	
**Season of conception**	17,060		13,334		3726		
January–March		9096 (53.3)		6694 (50.2)		2402 (64.5)	<0.001
April–June		323 (1.9)		263 (2.0)		60 (1.6)	
July–September		3136 (18.4)		2674 (20.1)		462 (12.4)	
October–December		4505 (26.4)		3703 (27.8)		802 (21.5)	
**Gestational duration** (weeks)	17,060	40.0 ± 1.2	13,334	40.0 ± 1.2	3726	39.8 ± 1.3	<0.001
**Sex of infant**	17,102		13,334		3768		
Boy		8770 (51.3)		6915 (51.9)		1855 (49.2)	0.004
Girl		8332 (48.7)		6419 (48.1)		1913 (50.8)	
**Term Birth weight** (g; at ≥ 37 weeks)	16,849	3353 ± 459	13,334	3364 ± 450	3515	3313 ± 490	<0.001

^1^ *p*-value from chi-squared test or *t*-test comparing included and excluded populations.

**Table 2:** Characteristics of the whole ELFE cohort (*n* = 17,358), and of the subpopulations included (*n* = 13,334) and excluded (*n* = 4024) from this study (all groups were restricted to term births, our target population).

Pollutant and Exposure Window				Percentiles					
	*n*	Mean	sd	p5	p25	p50	p75	p95	IQR
**PM_2.5_**									
Whole pregnancy	13,334	17.25	2.87	12.92	15.29	16.93	19.03	22.54	3.74
Whole pregnancy, truncated ^1^	13,334	17.25	3.16	12.51	15.02	16.92	19.21	22.96	4.18
1st trimester	13,334	18.87	5.56	10.89	14.69	18.06	22.41	29.16	7.71
2nd trimester	13,334	16.65	6.49	8.93	11.56	14.69	20.88	29.24	9.32
3rd trimester	13,334	16.15	5.74	9.65	12.10	14.49	19.24	28.04	7.14
3rd trimester, truncated ^1^	13,334	15.74	5.86	9.04	11.67	14.30	17.70	28.19	6.03
30 days before delivery	13,334	17.12	7.64	8.77	10.75	14.66	22.36	31.70	11.61
60 days before delivery	13,334	16.01	6.54	9.14	10.72	14.05	19.95	29.13	9.22
90 days before delivery	13,334	15.86	5.33	9.58	12.16	14.44	18.70	26.86	6.54
**PM_10_**									
Whole pregnancy	13,334	24.45	3.40	19.33	21.93	24.11	26.79	30.44	4.86
Whole pregnancy, truncated ^1^	13,334	24.42	3.63	18.95	21.79	24.13	26.86	30.86	5.07
1st trimester	13,334	25.95	6.21	17.35	21.12	24.97	30.14	37.10	9.02
2nd trimester	13,334	23.70	6.85	14.98	18.40	22.15	27.97	36.97	9.57
3rd trimester	13,334	23.65	6.20	15.54	19.17	22.11	27.42	36.12	8.25
3rd trimester, truncated ^1^	13,334	23.22	6.56	14.76	18.50	21.76	26.91	36.04	8.40
30 days before delivery	13,334	24.50	8.10	14.91	17.58	22.61	30.31	39.82	12.73
60 days before delivery	13,334	23.38	6.84	15.43	18.06	21.49	27.70	37.27	9.64
90 days before delivery	13,334	23.35	5.80	15.53	19.20	21.94	26.98	34.88	7.78
**NO_2_**									
Whole pregnancy	13,334	20.01	8.89	7.71	12.82	18.85	26.22	36.15	13.40
Whole pregnancy, truncated ^1^	13,334	19.98	9.01	7.57	12.75	18.70	26.25	36.44	13.50
1st trimester	13,334	22.08	10.34	7.44	14.05	20.70	28.88	41.17	14.83
2nd trimester	13,334	19.06	10.57	5.57	10.76	16.97	25.39	39.98	14.63
3rd trimester	13,334	18.80	10.06	5.81	10.98	16.95	25.00	38.86	14.02
3rd trimester, truncated ^1^	13,334	18.24	10.03	5.51	10.32	16.29	24.34	38.23	14.02
30 days before delivery	13,334	19.80	10.93	5.45	11.04	17.95	26.99	40.68	15.95
60 days before delivery	13,334	18.74	10.17	5.54	10.76	16.92	25.07	38.42	14.31
90 days before delivery	13,334	18.61	9.82	5.83	10.91	16.82	24.91	38.05	14.00

^1^ exposure truncated at 37 gestational weeks. IQR: interquartile range.

**Table 3:** Associations between atmospheric pollutant exposure during pregnancy and term birth weight, stratified according to the urbanization level. All effect estimates are reported for an increase by 5 µg/m^3^ for PM_2.5_ and PM_10_ and by 10 µg/m^3^ for NO_2_. Regression coefficients are estimated from weighted doubly robust generalized additive models ^1^ trimmed at the 1st and 99th percentiles (*n* = 13,334 pregnant women from ELFE cohort).

	Large City-Center Area (*n* = 2266)		Suburban Areas (*n* = 7331)		Rural Areas (*n* = 3737)		*p*-Value ^2^
	*β*	(95% CI)	*β*	(95% CI)	*β*	(95% CI)	
**PM_2.5_**							
Whole pregnancy	40.8	(8.32, 73.36)	7.4	(−15.45, 30.26)	3.9	(−30.66, 38.45)	0.120
Whole pregnancy, truncated ^3^	31.9	(2.78, 61.07)	5.7	(−14.74, 26.19)	12.1	(−18.63, 42.78)	0.241
1st trimester	38.1	(22.74, 53.46)	38.1	(27.56, 48.53)	68.0	(52.42, 83.67)	0.001
2nd trimester	−7.5	(−22.02, 6.97)	−9.6	(−20.05, 0.91)	−19.3	(−34.20, −4.44)	0.326
3rd trimester	−9.8	(−25.37, 5.71)	−25.1	(−35.39, −14.75)	−46.8	(−62.31, −31.29)	0.001
3rd trimester, truncated ^3^	−30.0	(−45.61, −14.45)	−45.6	(−56.37, −34.76)	−69.3	(−84.86, −53.8)	0.000
30 days before delivery	8.9	(−2.41, 20.22)	−1.9	(−9.10, 5.26)	−9.0	(−20.32, 2.37)	0.062
60 days before delivery	2.4	(−11.08, 15.87)	−8.4	(−17.26, 0.41)	−21.4	(−34.87, −7.87)	0.026
90 days before delivery	−3.1	(−19.94, 13.71)	−15.5	(−26.62, −4.35)	−36.3	(−52.94, −19.65)	0.007
**PM_10_**							
Whole pregnancy	32.6	(8.70, 56.54)	7.6	(−11.60, 26.87)	−9.1	(−40.14, 22.04)	0.058
Whole pregnancy, truncated ^3^	29.7	(7.08, 52.21)	6.9	(−10.49, 24.33)	0.7	(−26.81, 28.30)	0.141
1st trimester	37.8	(24.11, 51.57)	35.2	(25.43, 45.03)	59.0	(44.59, 73.46)	0.007
2nd trimester	0.0	(−13.17, 13.13)	−6.2	(−15.78, 3.36)	−15.4	(−28.80, −2.09)	0.172
3rd trimester	−5.1	(−18.92, 8.70)	−21.4	(−31.15, −11.68)	−47.9	(−62.09, −33.67)	0.000
3rd trimester, truncated ^3^	−23.1	(−36.73, −9.49)	−37.9	(−47.88, −27.84)	−64.1	(−77.95, −50.24)	0.000
30 days before delivery	9.9	(−0.58, 20.33)	−0.9	(−7.83, 6.05)	−8.8	(−19.44, 1.81)	0.031
60 days before delivery	3.7	(−8.98, 16.37)	−7.5	(−16.12, 1.17)	−24.0	(−36.57, −11.44)	0.003
90 days before delivery	0.8	(−13.87, 15.45)	−11.7	(−22.14, −1.32)	−38.0	(−53.31, −22.66)	0.000
**NO_2_**							
Whole pregnancy	32.7	(7.99, 57.34)	−7.7	(−22.23, 6.91)	−0.3	(−33.39, 32.76)	0.010
Whole pregnancy, truncated ^3^	31.0	(7.20, 54.81)	−7.6	(−21.91, 6.77)	1.8	(−29.84, 33.52)	0.011
1st trimester	37.8	(18.74, 56.83)	12.9	(0.83, 24.96)	46.3	(20.67, 71.98)	0.007
2nd trimester	0.5	(−17.84, 18.79)	−15.0	(−27.71, −2.35)	−23.6	(−49.27, 2.04)	0.164
3rd trimester	9.5	(−9.09, 28.03)	−12.8	(−24.81, −0.86)	−37.6	(−65.10, −10.08)	0.010
3rd trimester, truncated ^3^	−2.2	(−20.43, 15.98)	−20.3	(−32.37, −8.26)	−56.6	(−83.53, −29.70)	0.002
30 days before delivery	22.5	(5.57, 39.47)	−0.8	(−11.73, 10.11)	0.1	(−23.40, 23.64)	0.043
60 days before delivery	14.0	(−4.49, 32.56)	−6.0	(−17.71, 5.71)	−18.3	(−44.47, 7.94)	0.066
90 days before delivery	12.3	(−6.88, 31.44)	−7.8	(−20.02, 4.42)	−28.3	(−56.58, −0.01)	0.037

^1^ using the stabilized inverse probability to be exposed to air pollutants calculated using maternal education, in relationship status, parity, sex of the child, maternal active smoking during pregnancy, social deprivation, maternal French citizenship, maternal age, weight before pregnancy and maternal height, and further adjusted for the aforementioned covariates plus urbanization level, season of conception and an interaction term between the urbanization level and air pollutants. ^2^ *p*-value of the interaction test between the urbanization level and air pollutants. ^3^ exposure truncated at 37 gestational weeks.

## Data Availability

The data presented in this study are available on request from the corresponding author. The data are not publicly available due to privacy restrictions.

## Supplementary Materials

The following are available online at https://www.mdpi.com/article/10.3390/ijerph18115806/s1, Figure S1: Maternal exposure levels for each pollutant at each time windows considered in the study and Pearson’s correlation (*n* = 13,334 pregnant women from ELFE cohort), Figure S2: Association between atmospheric pollutant exposure during pregnancy and term birth weight accounting for gestational duration (*n* = 13,334 pregnant women from ELFE cohort), Figure S3: Association between atmospheric pollutant exposures during pregnancy and term birth weight using the minimum set of covariates in the propensity score model (*n* = 13,334 pregnant women from ELFE cohort), Table S1: Distribution of the weights used for the weighted generalized additive models (*n* = 13,334 pregnant women from ELFE cohort), Table S2: Association between atmospheric pollutant exposure during pregnancy and term birth weight (*n* = 13,334 pregnant women from ELFE cohort), Table S3: Association between atmospheric pollutant exposure during pregnancy and term birth weight with weighted doubly robust generalized additive models (*n* = 13,334 pregnant women from ELFE cohort). Table S4: Adjusted difference in mean term birth weight (g) associated with atmospheric pollutant exposures during pregnancy (*n* = 13,334 pregnant women from ELFE cohort) accounting for the correlation among trimesters of pregnancy, Table S5: Association between atmospheric pollutant exposure during pregnancy and term birth weight according to the deprivation index (EDI).
