# Supplementary material for: Maternal Ambient Exposure to Atmospheric Pollutants during Pregnancy and Offspring Term Birth Weight in the Nationwide ELFE Cohort
Source: Int J Environ Res Public Health. 2021 May 28;18(11):5806. doi: 10.3390/ijerph18115806 (PMC8198942; doi:10.3390/ijerph18115806)
Supplement: Supplementary file 1 [file ijerph-18-05806-s001.zip › ijerph-1220924-supplementary.pdf]

**Maternal ambient exposure to atmospheric pollutants during pregnancy and offspring term birth weight in the nation-wide ELFE cohort**

SUPPLEMENTAL MATERIAL

Equation 1: Propensity score of exposure used in the main analysis

$$PS_1 = E(Exposure) = \beta_1 education + \beta_2 relationship + \beta_3 parity + \beta_4 sex + \beta_5 smoking + \beta_6 deprivation + \beta_7 citizenship + \beta_8 heigth + \beta_9 age + \beta_{10} weigth + \varepsilon$$

Equation 2: Stabilized inverse probability weights

$$SW_i = \frac{Exposure}{PS_i}$$

Equation 3: Generalized additive model (outcome regression)

$$Y(TBW) = \{\beta_1 Exposure + \beta_2 urbanization + \beta_3 season + \beta_3 s(x,y)\}, SW$$

Equation 4: Propensity score of exposure used in the sensitivity analysis 1

$$PS_2 = E(Exposure) = \beta_1 education + \beta_2 relationship + \beta_3 parity + \beta_4 sex + \beta_5 smoking + \beta_6 deprivation + \beta_7 citizenship + \beta_8 heigth + \beta_9 age + \beta_{10} weigth + \beta_{11} gestational\ duration + \varepsilon$$

Equation 5: Propensity score of exposure used in the sensitivity analysis 2

$$PS_3 = E(Exposure) = \beta_1 education + \beta_2 deprivation + \beta_3 citizenship + \beta_4 parity + \varepsilon$$

Equation 6: Doubly robust GAM (outcome regression)

$$Y(TBW) = \{\beta_1 Exposure + \beta_2 education + \beta_3 relationship + \beta_4 parity + \beta_5 sex + \beta_6 smoking + \beta_7 deprivation + \beta_8 citizenship + \beta_9 heigth + \beta_{10} age + \beta_{11} weigth + \beta_{12} urbanization + \beta_{13} season + \beta_{14} s(x,y)\}, SW$$

Table S1: Distribution of the weights used for the weighted generalized additive models (n = 13,334 pregnant women from ELFE cohort).

| | $PM_{2.5}$ | | | | $PM_{10}$ | | | | $NO_2$ | | | |
|---|---|---|---|---|---|---|---|---|---|---|---|---|
| | Mean | Min | Max | Sum | Mean | Min | Max | Sum | Mean | Min | Max | Sum |
| **Weights[a]** | | | | | | | | | | | | |
| Whole pregnancy | 1.00 | 0.20 | 5.04 | 13,334.3 | 1.00 | 0.19 | 7.88 | 13,360.0 | 1.00 | 0.10 | 29.37 | 13,328.8 |
| Whole pregnancy truncated[b] | 1.00 | 0.20 | 4.21 | 13,334.5 | 1.00 | 0.19 | 5.92 | 13,340.5 | 1.00 | 0.09 | 31.03 | 13,340.8 |
| 1st trimester | 1.00 | 0.36 | 2.14 | 13,337.4 | 1.00 | 0.33 | 3.16 | 13,336.8 | 1.00 | 0.12 | 15.05 | 13,314.5 |
| 2nd trimester | 1.00 | 0.32 | 2.00 | 13,334.7 | 1.00 | 0.27 | 2.45 | 13,329.5 | 1.00 | 0.09 | 11.70 | 13,284.5 |
| 3rd trimester | 1.00 | 0.28 | 2.08 | 13,332.0 | 1.00 | 0.15 | 3.05 | 13,331.7 | 1.00 | 0.08 | 18.16 | 13,303.5 |
| 3rd trimester truncated[b] | 1.00 | 0.25 | 2.16 | 13,332.3 | 1.00 | 0.15 | 2.95 | 13,333.1 | 1.00 | 0.08 | 16.66 | 13,307.0 |
| 30 days before delivery | 1.00 | 0.46 | 2.24 | 13,334.3 | 1.00 | 0.34 | 2.34 | 13,334.3 | 1.00 | 0.13 | 9.73 | 13,309.9 |
| 60 days before delivery | 1.00 | 0.40 | 1.93 | 13,332.7 | 1.00 | 0.18 | 2.56 | 13,330.6 | 1.00 | 0.09 | 16.90 | 13,301.0 |
| 90 days before delivery | 1.00 | 0.35 | 2.21 | 13,331.6 | 1.00 | 0.17 | 3.14 | 13,331.2 | 1.00 | 0.09 | 21.40 | 13,307.7 |
| **Weights[c] with gestational duration** | | | | | | | | | | | | |
| Whole pregnancy | 1.00 | 0.21 | 5.68 | 13,332.6 | 1.00 | 0.20 | 7.70 | 13,363.5 | 1.00 | 0.10 | 28.65 | 13,329.9 |
| Whole pregnancy truncated[b] | 1.00 | 0.21 | 5.21 | 13,331.7 | 1.00 | 0.19 | 6.23 | 13,340.8 | 1.00 | 0.09 | 31.46 | 13,338.1 |
| 1st trimester | 1.00 | 0.29 | 2.90 | 13,342.3 | 1.00 | 0.22 | 3.86 | 13,346.5 | 1.00 | 0.12 | 14.77 | 13,315.7 |
| 2nd trimester | 1.00 | 0.35 | 2.12 | 13,333.7 | 1.00 | 0.29 | 2.58 | 13,328.5 | 1.00 | 0.09 | 12.82 | 13,279.0 |
| 3rd trimester | 1.00 | 0.24 | 2.39 | 13,333.5 | 1.00 | 0.12 | 3.28 | 13,328.4 | 1.00 | 0.08 | 24.40 | 13,302.9 |
| 3rd trimester truncated[b] | 1.00 | 0.21 | 2.67 | 13,334.2 | 1.00 | 0.13 | 3.38 | 13,326.4 | 1.00 | 0.08 | 23.09 | 13,307.0 |
| 30 days before delivery | 1.00 | 0.48 | 2.25 | 13,334.9 | 1.00 | 0.33 | 2.36 | 13,334.8 | 1.00 | 0.13 | 11.28 | 13,309.8 |
| 60 days before delivery | 1.00 | 0.40 | 1.98 | 13,333.1 | 1.00 | 0.18 | 2.65 | 13,330.6 | 1.00 | 0.09 | 20.48 | 13,302.3 |
| 90 days before delivery | 1.00 | 0.34 | 2.28 | 13,331.9 | 1.00 | 0.17 | 3.22 | 13,330.7 | 1.00 | 0.09 | 26.51 | 13,310.4 |
| **Weights[d] with the minimal set of covariates** | | | | | | | | | | | | |
| Whole pregnancy | 1.00 | 0.34 | 3.95 | 13,335.1 | 1.00 | 0.25 | 7.22 | 13,368.4 | 1.00 | 0.16 | 24.29 | 13,306.9 |
| Whole pregnancy truncated[b] | 1.00 | 0.35 | 3.16 | 13,334.2 | 1.00 | 0.25 | 5.40 | 13,347.9 | 1.00 | 0.15 | 25.19 | 13,320.7 |
| 1st trimester | 1.00 | 0.46 | 2.15 | 13,336.9 | 1.00 | 0.38 | 3.37 | 13,337.8 | 1.00 | 0.17 | 13.71 | 13,315.2 |
| 2nd trimester | 1.00 | 0.60 | 1.68 | 13,333.7 | 1.00 | 0.42 | 2.05 | 13,330.3 | 1.00 | 0.20 | 10.49 | 13,292.7 |
| 3rd trimester | 1.00 | 0.45 | 2.01 | 13,333.4 | 1.00 | 0.22 | 2.77 | 13,333.5 | 1.00 | 0.15 | 16.84 | 13,282.7 |
| 3rd trimester truncated[b] | 1.00 | 0.47 | 1.94 | 13,333.4 | 1.00 | 0.21 | 2.56 | 13,333.9 | 1.00 | 0.14 | 13.73 | 13,287.2 |
| 30 days before delivery | 1.00 | 0.54 | 1.94 | 13,334.2 | 1.00 | 0.44 | 1.97 | 13,334.6 | 1.00 | 0.20 | 9.89 | 13,297.0 |
| 60 days before delivery | 1.00 | 0.51 | 1.85 | 13,333.8 | 1.00 | 0.27 | 2.49 | 13,332.5 | 1.00 | 0.16 | 12.17 | 13,284.2 |
| 90 days before delivery | 1.00 | 0.49 | 1.97 | 13,333.4 | 1.00 | 0.24 | 2.73 | 13,332.9 | 1.00 | 0.15 | 12.25 | 13,281.5 |

[a] using the stabilized inverse probability to be exposed to air pollutants calculated using maternal education, in relationship status, parity, sex of the child, maternal active smoking during pregnancy, social deprivation, maternal French citizenship, maternal age, weight before pregnancy and maternal height.

[b] exposure truncated at 37 gestational weeks.

[c] using the stabilized inverse probability to be exposed to air pollutants calculated using maternal education, in relationship status, parity, sex of the child, maternal active smoking during pregnancy, social deprivation, maternal French citizenship, maternal age, weight before pregnancy, maternal height and gestational duration.

[d] using the stabilized inverse probability to be exposed to air pollutants calculated using maternal education, parity, social deprivation and maternal French citizenship.

Table S2: Association between atmospheric pollutant exposure during pregnancy and term birth weight (n = 13,334 pregnant women from ELFE cohort). All effect estimates correspond to an increase of 5 µg/m$^3$ for $PM_{2.5}$ and $PM_{10}$ and 10 µg/m$^3$ for $NO_2$. Regression coefficients are from weighted generalized additive models[a].

| | Full weights | | Trimmed weights | |
|---|---|---|---|---|
| | $\beta$ | (95% CI) | $\beta$ | (95% CI) |
| **$PM_{2.5}$** | | | | |
| Whole pregnancy | 6.53 | (-15.01, 28.08) | 7.55 | (-14.05, 29.16) |
| Whole pregnancy | 5.03 | (-14.29, 24.34) | 5.79 | (-13.57, 25.15) |
| 1st trimester | 40.96 | (31.51, 50.41) | 40.82 | (31.38, 50.26) |
| 2nd trimester | -12.33 | (-22.45, -2.22) | -12.48 | (-22.59, -2.37) |
| 3rd trimester | -26.98 | (-36.60, -17.37) | -26.87 | (-36.47, -17.26) |
| 3rd trimester truncated[b] | -48.32 | (-58.58, -38.05) | -48.38 | (-58.63, -38.12) |
| 30 days before delivery | -1.99 | (-8.49, 4.51) | -1.69 | (-8.19, 4.81) |
| 60 days before delivery | -9.15 | (-17.31, -0.98) | -8.92 | (-17.08, -0.77) |
| 90 days before delivery | -17.65 | (-28.03, -7.28) | -17.63 | (-27.99, -7.26) |
| **$PM_{10}$** | | | | |
| Whole pregnancy | 9.68 | (-7.31, 26.67) | 11.79 | (-4.56, 28.76) |
| Whole pregnancy | 9.08 | (-6.01, 24.18) | 38.37 | (-3.29, 26.87) |
| 1st trimester | 38.43 | (29.87, 46.99) | -7.10 | (29.81, 46.94) |
| 2nd trimester | -6.92 | (-15.90, 2.05) | -26.91 | (-16.08, 1.89) |
| 3rd trimester | -26.95 | (-36.25, -17.65) | -42.38 | (-36.20, -17.63) |
| 3rd trimester truncated[b] | -41.75 | (-51.16, -32.34) | -0.07 | (-51.80, -32.97) |
| 30 days before delivery | -0.39 | (-6.57, 5.80) | -8.08 | (-6.26, 6.11) |
| 60 days before delivery | -8.09 | (-15.95, -0.24) | -13.98 | (-15.93, -0.23) |
| 90 days before delivery | -13.75 | (-23.33, -4.18) | -0.21 | (-23.54, -4.42) |
| **$NO_2$** | | | | |
| Whole pregnancy | 8.97 | (-4.73, 22.67) | -0.35 | (-14.09, 13.68) |
| Whole pregnancy | 9.11 | (-4.27, 22.49) | 20.19 | (-14.04, 13.34) |
| 1st trimester | 20.93 | (9.90, 31.95) | -12.86 | (9.13, 31.25) |
| 2nd trimester | -8.93 | (-21.29, 3.43) | -10.39 | (-25.41, -0.32) |
| 3rd trimester | -10.21 | (-22.20, 1.79) | -27.21 | (-21.76, 0.98) |
| 3rd trimester truncated[b] | -24.33 | (-36.61, -12.05) | 4.91 | (-39.60, -14.81) |
| 30 days before delivery | 6.21 | (-3.93, 16.35) | -2.76 | (-5.31, 15.31) |
| 60 days before delivery | 1.15 | (-9.79, 12.10) | -5.23 | (-13.81, 8.30) |
| 90 days before delivery | -0.78 | (-12.23, 10.67) | -0.35 | (-16.76, 6.29) |

[a] using the stabilized inverse probability to be exposed to air pollutants calculated using maternal education, in relationship status, parity, sex of the child, maternal active smoking during pregnancy, social deprivation, maternal French citizenship, maternal age, weight before pregnancy, maternal height and further adjusted for urbanization level and season of conception.

[b] exposure truncated at 37 gestational weeks.

Table S3: Association between atmospheric pollutant exposure during pregnancy and term birth weight (n = 13,334 pregnant women from ELFE cohort). All effect estimates correspond to an increase of 5 µg/m$^3$ for $PM_{2.5}$ and $PM_{10}$ and 10 µg/m$^3$ for $NO_2$. Regression coefficients are from weighted doubly robust generalized additive models[a].

| | Full weights | Trimmed weights |
|---|---|---|
| | $\beta$ (95% CI) | $\beta$ (95% CI) |
| **$PM_{2.5}$** | | |
| Whole pregnancy | 14.66 (-5.64, 34.96) | 14.59 (-5.72, 34.91) |
| Whole pregnancy | 12.86 (-5.30, 31.01) | 12.68 (-5.49, 30.86) |
| 1st trimester | 44.04 (35.13, 52.95) | 43.83 (34.94, 52.72) |
| 2nd trimester | -10.35 (-19.83, -0.87) | -10.64 (-20.11, -1.17) |
| 3rd trimester | -25.56 (-34.58, -16.55) | -25.58 (-34.58, -16.57) |
| 3rd trimester truncated[b] | -46.11 (-55.72, -36.50) | -46.19 (-55.79, -36.58) |
| 30 days before delivery | -1.01 (-7.10, 5.09) | -0.97 (-7.07, 5.13) |
| 60 days before delivery | -8.47 (-16.12, -0.81) | -8.42 (-16.07, -0.77) |
| 90 days before delivery | -16.41 (-26.13, -6.68) | -16.53 (-26.24, -6.81) |
| **$PM_{10}$** | | |
| Whole pregnancy | 13.46 (-3.46, 30.38) | 14.39 (-2.14, 30.92) |
| Whole pregnancy | 11.27 (-3.67, 26.21) | 13.09 (-1.71, 27.88) |
| 1st trimester | 40.46 (32.14, 48.79) | 40.27 (31.94, 48.59) |
| 2nd trimester | -6.27 (-14.64, 2.10) | -6.43 (-14.80, 1.95) |
| 3rd trimester | -22.31 (-30.63, -13.99) | -22.46 (-30.77, -14.14) |
| 3rd trimester truncated[b] | -39.02 (-47.85, -30.18) | -39.67 (-48.50, -30.83) |
| 30 days before delivery | 0.05 (-5.74, 5.84) | -0.01 (-5.80, 5.78) |
| 60 days before delivery | -8.10 (-15.44, -0.76) | -8.21 (-15.54, -0.88) |
| 90 days before delivery | -12.90 (-21.76, -4.05) | -13.31 (-22.17, -4.46) |
| **$NO_2$** | | |
| Whole pregnancy | 8.82 (-4.13, 21.78) | 0.54 (-12.55, 13.64) |
| Whole pregnancy | 10.08 (-2.64, 22.80) | 0.66 (-12.25, 13.57) |
| 1st trimester | 22.60 (11.90, 33.31) | 21.63 (10.89, 32.36) |
| 2nd trimester | -7.49 (-18.92, 3.94) | -11.76 (-23.38, -0.15) |
| 3rd trimester | -6.62 (-16.98, 3.75) | -9.51 (-20.00, 0.98) |
| 3rd trimester truncated[b] | -15.94 (-26.41, -5.48) | -18.59 (-29.17, -8.01) |
| 30 days before delivery | 5.83 (-3.65, 15.32) | 4.62 (-4.95, 14.19) |
| 60 days before delivery | 0.55 (-9.64, 10.74) | -2.55 (-12.85, 7.75) |
| 90 days before delivery | -1.28 (-11.85, 9.29) | -4.75 (-15.45, 5.94) |

[a] using the stabilized inverse probability to be exposed to air pollutants calculated using maternal education, in relationship status, parity, sex of the child, maternal active smoking during pregnancy, social deprivation, maternal French citizenship, maternal age, weight before pregnancy, maternal height and further adjusted for the aforementionned covariates plus urbanization level and season of conception.
[b] exposure truncated at 37 gestational weeks.

Table S4: Adjusted difference in mean term birth weight (g) associated with atmospheric pollutant exposures during pregnancy (n = 13,334 pregnant women from ELFE cohort) accounting for the correlation among trimesters of pregnancy. All effect estimates correspond to an increase of 5 µg/$m^3$ for $PM_{2.5}$ and $PM_{10}$ and 10 µg/$m^3$ for $NO_2$. Regression coefficients are from weighted doubly robust generalized additive models[a].

| | Full weights | Trimmed weights |
|---|---|---|
| | *β* (95% CI) | *β* (95% CI) |
| **$PM_{2.5}$** | | |
| 1st trimester | 45.92 (36.92, 54.92) | 45.71 (36.72, 54.69) |
| 2nd trimester | -17.06 (-26.78, -7.34) | -17.33 (-27.04, -7.61) |
| 3rd trimester | -25.44 (-34.77, -16.11) | -25.47 (-34.79, -16.15) |
| 3rd trimester truncated[b] | -48.08 (-58.14, -38.01) | -48.17 (-58.23, -38.11) |
| **$PM_{10}$** | | |
| 1st trimester | 42.32 (34.03, 50.61) | 42.15 (33.85, 50.45) |
| 2nd trimester | -18 (-26.61, -9.39) | -18.14 (-26.75, -9.52) |
| 3rd trimester | -24.31 (-32.52, -16.09) | -24.37 (-32.59, -16.16) |
| 3rd trimester truncated[b] | -38.13 (-46.65, -29.61) | -38.6 (-47.14, -30.06) |
| **$NO_2$** | | |
| 1st trimester | 18.36 (7.60, 29.13) | 17.14 (6.37, 27.91) |
| 2nd trimester | -15.92 (-27.63, -4.22) | -20.04 (-31.93, -8.16) |
| 3rd trimester | -4.09 (-14.69, 6.50) | -7.26 (-18.01, 3.48) |
| 3rd trimester truncated[b] | -14.65 (-25.33, -3.97) | -17.66 (-28.50, -6.82) |

[a] using the stabilized inverse probability to be exposed to air pollutants calculated using maternal education, in relationship status, parity, sex of the child, maternal active smoking during pregnancy, social deprivation, maternal French citizenship, maternal age, weight before pregnancy, maternal height and further adjusted for the aforementionned covariates plus urbanization level, season of conception and residuals of the regression between trimesters.

[b] exposure truncated at 37 gestational weeks.

Table S5: Association between atmospheric pollutant exposure during pregnancy and term birth weight according to the deprivation index (EDI). All effect estimates correspond to an increase of 5 µg/m$^3$ for $PM_{2.5}$ and $PM_{10}$ and 10 µg/m$^3$ for $NO_2$. Regression coefficients are from weighted doubly robust generalized additive models[a] trimmed at the 1$^{st}$ and 99$^{th}$ percentiles (n = 13,334 pregnant women from ELFE cohort).

| | **EDI Q1** (least deprived) (n=2,287) | **EDI Q2** (n=2,383) | **EDI Q3** (n=2,166) | **EDI Q4** (n=2,499) | **EDI Q5** (most deprived) (n=3,999) | p-value[b] |
|---|---|---|---|---|---|---|
| | $\beta$ (95% CI) | $\beta$ (95% CI) | $\beta$ (95% CI) | $\beta$ (95% CI) | $\beta$ (95% CI) | |
| **$PM_{2.5}$** | | | | | | |
| Whole pregnancy | 18.61 (-17.77, 55.00) | 22.57 (-12.31, 57.46) | 0.17 (-33.90, 34.23) | 2.89 (-29.41, 35.20) | 23.56 (-3.87, 50.99) | 0.652 |
| Whole pregnancy truncated[d] | 23.06 (-9.38, 55.50) | 26.96 (-4.56, 58.48) | 3.16 (-27.73, 34.05) | -0.90 (-30.30, 28.49) | 14.37 (-10.38, 39.11) | 0.545 |
| 1$^{st}$ trimester | 51.31 (33.86, 68.77) | 54.33 (37.49, 71.18) | 44.88 (27.59, 62.17) | 38.70 (22.92, 54.48) | 37.16 (24.07, 50.25) | 0.371 |
| 2$^{nd}$ trimester | -7.35 (-23.08, 8.39) | -9.59 (-25.42, 6.24) | -13.04 (-28.94, 2.85) | -20.37 (-35.21, -5.54) | -5.99 (-18.35, 6.37) | 0.484 |
| 3$^{rd}$ trimester | -37.60 (-54.39, -20.80) | -30.73 (-47.36, -14.09) | -40.02 (-57.16, -22.87) | -16.15 (-31.62, -0.68) | -16.70 (-29.55, -3.85) | 0.042 |
| 3$^{rd}$ trimester truncated[d] | -58.15 (-74.99, -41.31) | -50.84 (-67.41, -34.26) | -61.43 (-78.68, -44.18) | -38.55 (-54.14, -22.97) | -36.27 (-49.37, -23.16) | 0.031 |
| 30 days before delivery | -5.93 (-18.32, 6.46) | -5.71 (-18.14, 6.72) | -6.68 (-19.34, 5.98) | 8.34 (-2.95, 19.63) | 0.61 (-8.50, 9.73) | 0.261 |
| 60 days before delivery | -17.53 (-32.19, -2.87) | -11.44 (-26.08, 3.20) | -18.42 (-33.45, -3.39) | 1.71 (-11.77, 15.19) | -4.42 (-15.46, 6.63) | 0.135 |
| 90 days before delivery | -27.12 (-45.22, -9.02) | -20.21 (-38.07, -2.36) | -28.35 (-46.66, -10.05) | -8.26 (-24.95, 8.44) | -9.24 (-23.18, 4.69) | 0.195 |
| **$PM_{10}$** | | | | | | |
| Whole pregnancy | 36.39 (4.80, 67.98) | 36.87 (7.13, 66.62) | 16.97 (-12.26, 46.19) | -0.84 (-27.29, 25.61) | 6.25 (-15.30, 27.80) | 0.116 |
| Whole pregnancy truncated[d] | 33.25 (4.94, 61.56) | 34.44 (7.24, 61.65) | 16.93 (-10.05, 43.90) | -2.14 (-26.72, 22.43) | 3.97 (-16.13, 24.06) | 0.086 |
| 1$^{st}$ trimester | 47.47 (31.80, 63.14) | 47.79 (32.57, 63.01) | 41.29 (25.45, 57.14) | 38.25 (23.92, 52.58) | 33.16 (21.16, 45.16) | 0.416 |
| 2$^{nd}$ trimester | -2.12 (-16.58, 12.34) | -5.01 (-19.46, 9.43) | -6.58 (-21.19, 8.04) | -15.87 (-29.46, -2.28) | -3.49 (-14.91, 7.94) | 0.516 |
| 3$^{rd}$ trimester | -32.17 (-48.19, -16.14) | -22.95 (-37.99, -7.90) | -31.72 (-47.22, -16.21) | -18.61 (-32.73, -4.49) | -15.95 (-27.89, -4.00) | 0.287 |
| 3$^{rd}$ trimester truncated[d] | -49.58 (-65.07, -34.09) | -40.08 (-54.70, -25.45) | -48.19 (-63.46, -32.93) | -37.77 (-51.73, -23.80) | -32.12 (-44.11, -20.14) | 0.224 |
| 30 days before delivery | -1.43 (-13.25, 10.39) | -1.73 (-13.14, 9.69) | -3.91 (-15.70, 7.88) | 7.10 (-3.58, 17.78) | -0.95 (-9.74, 7.84) | 0.625 |
| 60 days before delivery | -14.08 (-28.26, 0.10) | -7.46 (-21.07, 6.15) | -14.97 (-28.97, -0.97) | -1.98 (-14.78, 10.83) | -6.46 (-17.27, 4.35) | 0.554 |
| 90 days before delivery | -18.98 (-36.22, -1.74) | -12.16 (-28.29, 3.97) | -19.31 (-35.81, -2.82) | -11.56 (-26.63, 3.50) | -9.79 (-22.50, 2.92) | 0.821 |
| **$NO_2$** | | | | | | |
| Whole pregnancy | 13.06 (-11.17, 37.29) | 18.19 (-3.38, 39.76) | -6.14 (-27.25, 14.96) | 0.52 (-19.55, 20.59) | -11.64 (-30.63, 7.34) | 0.133 |
| Whole pregnancy truncated[d] | 14.54 (-9.09, 38.17) | 19.19 (-1.95, 40.32) | -5.49 (-26.27, 15.29) | -0.97 (-20.85, 18.90) | -12.37 (-31.08, 6.34) | 0.082 |
| 1$^{st}$ trimester | 34.22 (14.21, 54.23) | 37.32 (19.27, 55.37) | 21.98 (3.60, 40.37) | 14.18 (-2.94, 31.29) | 11.04 (-4.70, 26.78) | 0.074 |
| 2$^{nd}$ trimester | -4.70 (-24.78, 15.37) | -1.85 (-20.53, 16.82) | -21.75 (-40.25, -3.24) | -13.69 (-31.32, 3.94) | -13.60 (-29.69, 2.48) | 0.439 |
| 3$^{rd}$ trimester | -7.03 (-28.00, 13.95) | -8.67 (-27.50, 10.15) | -19.28 (-36.96, -1.59) | 1.19 (-15.71, 18.09) | -12.71 (-28.66, 3.23) | 0.443 |
| 3$^{rd}$ trimester truncated[d] | -16.07 (-37.09, 4.95) | -19.13 (-37.98, -0.29) | -27.13 (-44.82, -9.45) | -9.85 (-26.87, 7.16) | -20.15 (-36.08, -4.22) | 0.631 |
| 30 days before delivery | 11.21 (-7.64, 30.05) | 2.47 (-14.86, 19.80) | -2.37 (-18.98, 14.25) | 18.18 (2.45, 33.91) | -2.94 (-17.45, 11.58) | 0.176 |
| 60 days before delivery | 3.34 (-17.17, 23.85) | -2.91 (-21.58, 15.75) | -12.08 (-29.70, 5.54) | 10.62 (-6.15, 27.40) | -8.75 (-24.41, 6.90) | 0.238 |
| 90 days before delivery | 1.45 (-19.99, 22.89) | -2.03 (-21.24, 17.18) | -13.29 (-31.32, 4.73) | 5.62 (-11.63, 22.88) | -11.27 (-27.57, 5.03) | 0.396 |

[a] using the stabilized inverse probability to be exposed to air pollutants calculated using maternal education, in relationship status, parity, sex of the child, maternal active smoking during pregnancy, social deprivation, maternal French citizenship, maternal age, weight before pregnancy, maternal height and further adjusted for the aforementionned covariates plus urbanization level, season of conception as well as an interaction term between European Deprivation Index and air pollutant.

[b] p-value of the interaction test between European Deprivation Index and air pollutant.

[c] exposure truncated at 37 gestational weeks.

Figure S1: Maternal exposure levels for each pollutant at each time windows considered in the study and Pearson's correlation (n = 13,334 pregnant women from ELFE cohort).

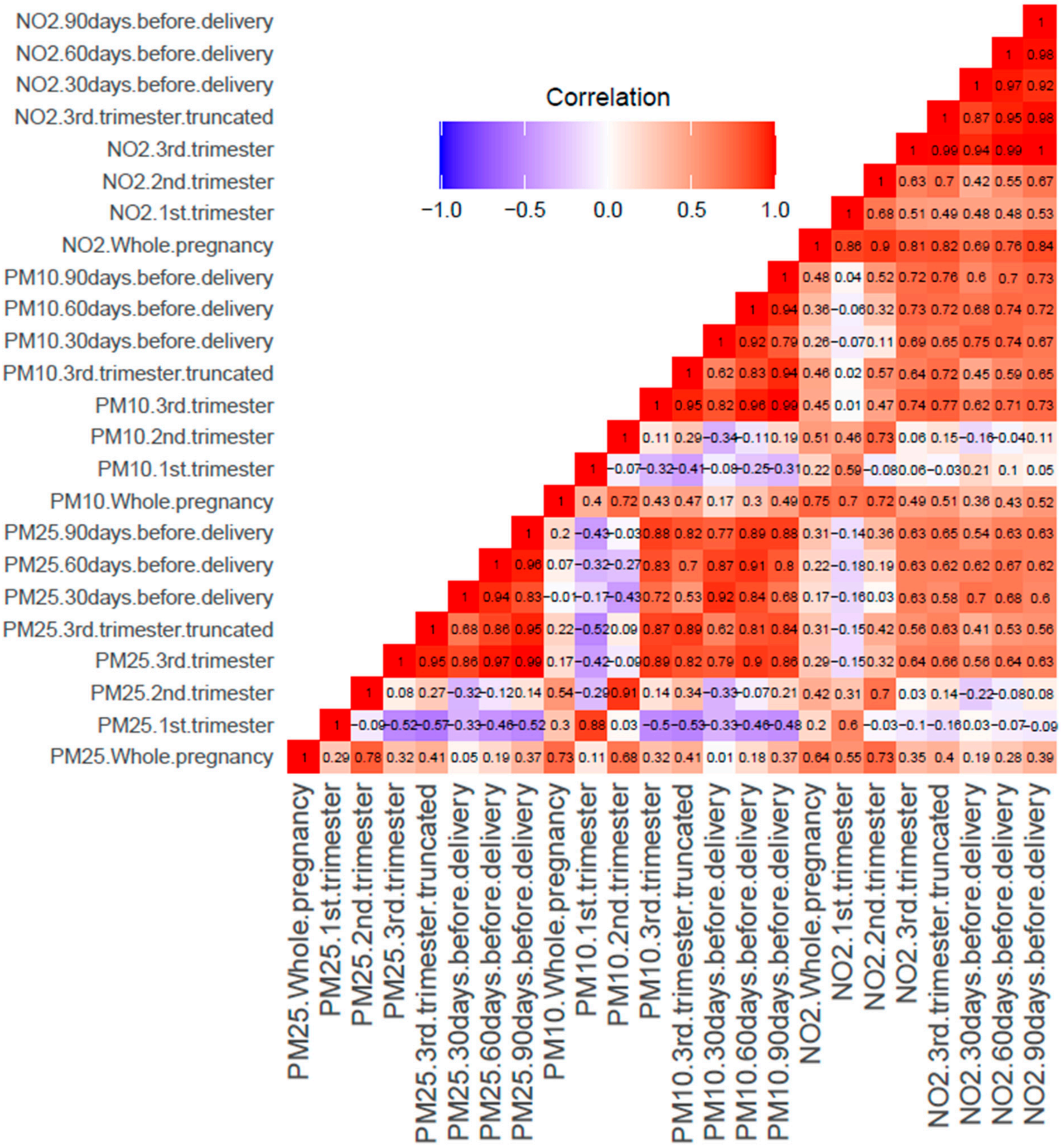

Figure S2: Association between atmospheric pollutant exposure during pregnancy and term birth weight accounting for gestational duration (n = 13,334 pregnant women from ELFE cohort). All effect estimates correspond to an increase of 5 µg/m³ for $PM_{2.5}$ and $PM_{10}$ and 10 µg/m³ for $NO_2$. Regression coefficients are from weighted generalized additive models[a]. Full weights are represented in black, and trimmed weights in grey. The point reflects the central estimate, the line represents the 95% confidence interval.

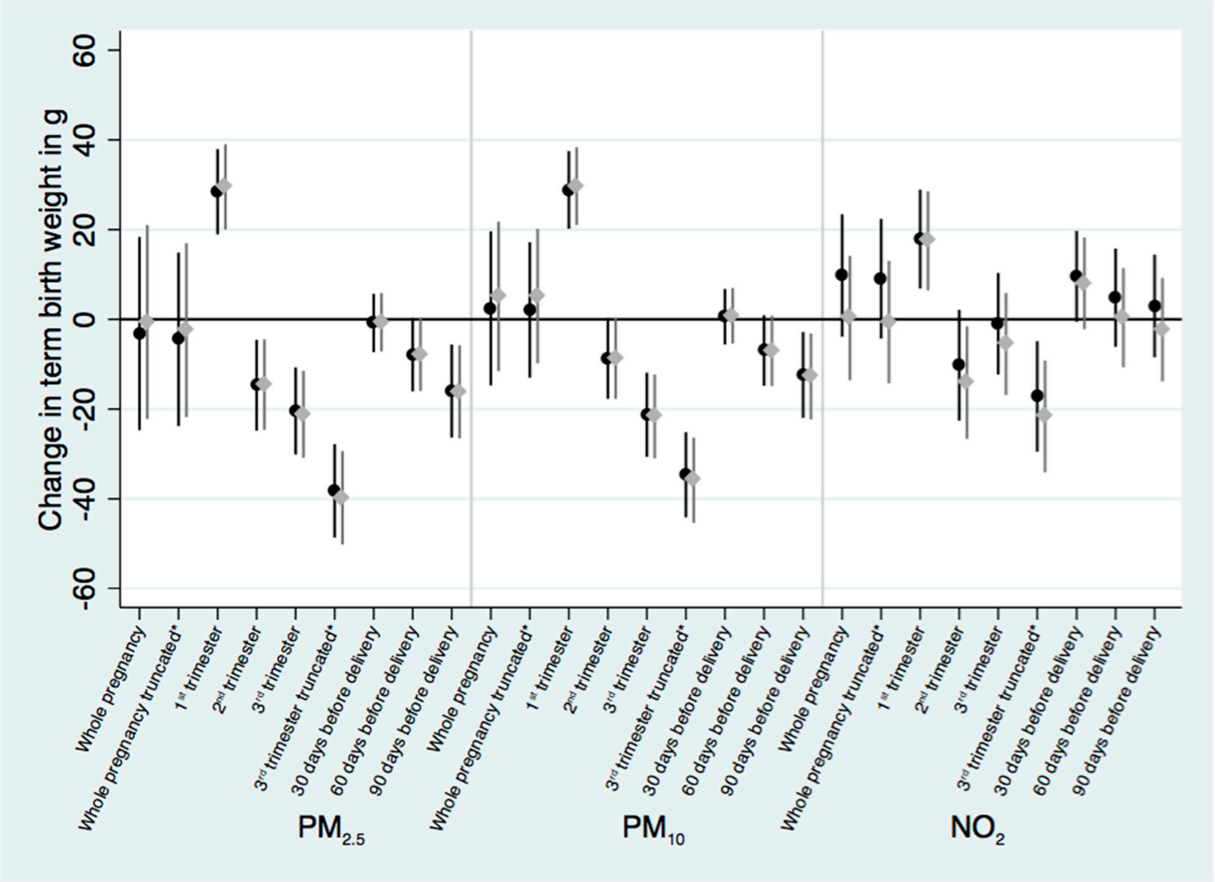


[a] using the stabilized inverse probability to be exposed to air pollutants calculated using maternal education, in relationship status, parity, sex of the child, maternal active smoking during pregnancy, social deprivation, maternal French citizenship, maternal age, weight before pregnancy, maternal height and gestational duration further adjusted for urbanization level and season of conception.

* exposure truncated at 37 gestational weeks.

Figure S3: Association between atmospheric pollutant exposures during pregnancy and term birth weight using the minimum set of covariates in the propensity score model (n = 13,334 pregnant women from ELFE cohort). All effect estimates correspond to an increase of 5 µg/m$^3$ for $PM_{2.5}$ and $PM_{10}$ and 10 µg/m$^3$ for $NO_2$. Regression coefficients are from weighted generalized additive models[a]. Full weights are represented in black and trimmed weights in grey. The point reflects the central estimate, the line represents the 95% confidence interval.

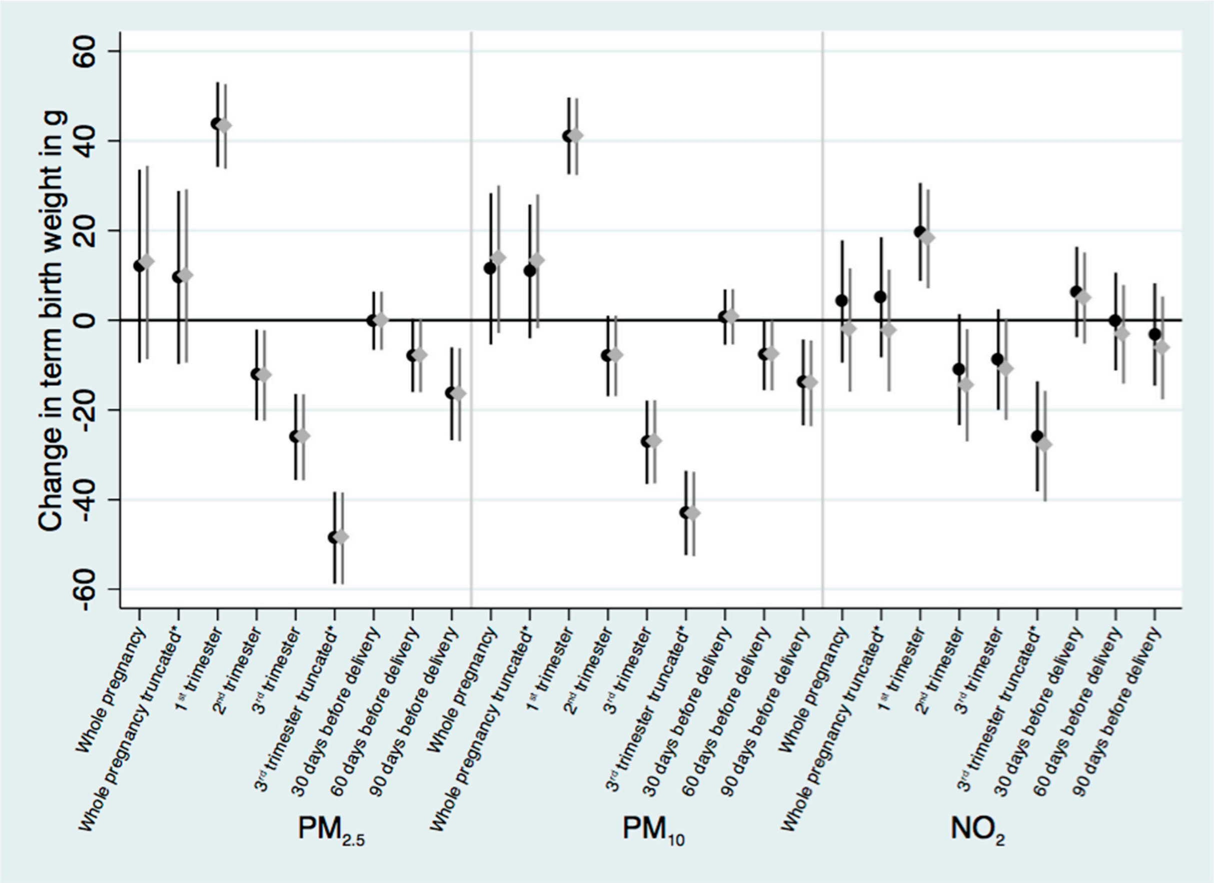


[a] using the stabilized inverse probability to be exposed to air pollutants calculated using maternal education, parity, social deprivation, maternal French citizenship and further adjusted for urbanization level and season of conception.

* exposure truncated at 37 gestational weeks.
